# The complete chloroplast genome of *Protea kilimandscharica* Engl. (Proteaceae)

**DOI:** 10.1080/23802359.2019.1710603

**Published:** 2020-01-14

**Authors:** John M. Nzei, Josphat K. Saina, Virginia M. Mwanzia, Sheila Avoga, Cheng Pan

**Affiliations:** a Sino-Africa Joint Research Center, Chinese Academy of Sciences, Wuhan, Hubei, China;; b University of Chinese Academy of Sciences, Beijing, China;; c CAS Key Laboratory of Plant Germplasm Enhancement and Specialty Agriculture, Wuhan Botanical Garden, The Innovative Academy of Seed Design, Chinese Academy of Sciences, Wuhan, China;; d Center of Economic Botany, Core Botanical Gardens, Chinese Academy of Sciences, Wuhan, China

**Keywords:** Proteaceae, *Protea kilimandscharica*, chloroplast genome, phylogenetic

## Abstract

*Protea kilimandscharica* is endemic to the heath zone of Mt Kenya, restricted to the rocky slopes of the mountain. The complete chloroplast genome of *P*. *kilimandscharica* was determined by next-generation sequencing technology, with a total length of 158,657 bp. The cp genome encodes 115 unique genes, with four rRNA genes, 81 protein-coding genes (PCGs), and 30 tRNA genes. A 3.1 kb inversion was noted in the LSC. Phylogenetic analysis, based on 75 common protein-coding genes revealed *P*. *kilimandscharica* as sister to *Macadamia integrifolia* and *Macadamia ternifolia*.

The species, *Protea kilimandscharica* is endemic to heath zone (3000–3800 m) and on rocky slopes of Mt Kenya in nutrient poor soils, recurrent fires and rocky habitats. *Protea kilimandscharica* is a perennial species among the 112 species forming the largest genus of the family Proteaceae (Rourke 1980), restricted to Southern Hemisphere and tropical regions of Africa (Galley and Linder 2006; Duchene and Bromham 2013). The genus consists of evergreen sclerophyllous shrubs, some growing into short trees exhibiting substantial diversity in leaf shape, size, and flower variation. Additionally, the species represent intraspecific variation within their habit and a higher rate of diversification than the rest of the Proteaceae family (Mitchell et al. 2017), and a rapid generation turnover and diversification under the influence of edaphic and fire pressure (Rourke 1980). In this research, the complete chloroplast genome of *P. kilimandscharica* was sequenced for the first time in order to provide useful genomic information to the chloroplast genome feature of the genus *Protea*, an additional resource to the family Proteaceae, useful in determining the general evolutionary mechanisms within the genus.

The leaf sample of *P*. *kilimandscharica* was obtained from Mt. Kenya (00°03′31″S; 37°17′33″E), and the voucher specimen was deposited at Wuhan Botanical Garden herbarium (SAJIT 20174). Total genomic DNA was extracted using a modified CTAB by Doyle (1991), then sequenced using Illumina Hiseq 2500 platform at NOVOgene Company (Beijing, China). *De novo* assembly produced a total of 5.2 Gb raw data which were filtered by PRINSEQ lite Ver0.20.4 and the high-quality reads (5 G) assembled by NOVOPlasty 2.7.0 with K-mer 39 using reference chloroplast genome of *Macadamia ternifolia* (MF678834). Gene annotation was done by chlorobox (Tillich et al. 2017), and manual amendments based on BLAST (https://blast.ncbi.nlm.nih.gov/Blast.cgi). Moreover, the tRNA genes were verified using tRNAscanSE 2.0 (http://lowelab.ucsc.edu/tRNAscan-SE/) and the annotated genome sequence was submitted to NCBI GenBank (Accession number MH362765).

The chloroplast genome of *P*. *kilimandscharica* was 158,657 bp in length, with a GC content of 38%. It comprised of two inverted repeat (IR; 26,389 bp) regions, a small single-copy (SSC; 18,535 bp) region, and a large single-copy (LSC; 87,344 bp) region with a minor inversion of 3.1 kb. The cp genome had 115 unique genes, including 81 protein-coding genes, 30 transfer RNA, and 4 ribosomal RNA genes. In addition, 13 genes had single introns, seven PCGs (*rpl2*, *rps16*, *rpoC1*, *ndhA*, *ndhB*, *petB*, *atpF*), and six tRNAs (*trnA*-*UGC*, *trnG*-*UCC*, *trnI*-*GAU*, *trnK*-*UUU*, *trnL*-*UAA*, *and trnV*-*UAC*), while three genes displayed two introns (*rps12*, *ycf3*, and *clpP*).

To assess the phylogenetic position of *Protea* in Proteales, eight chloroplast genomes including the outgroup (*Achyls triphylla*) were downloaded from NCBI. Then, 75 shared protein-coding genes were extracted from the nine chloroplast genomes and aligned using MAFFT v.7 (Katoh and Standley 2013). The maximum likelihood (ML) analysis was conducted using RAxML version 8.0.20 (Stamatakis 2014), with the best fitting substitution model (GTG + I + G) obtained from jModelTest v2.1.4 (Darriba et al. 2012). The ML tree revealed *P*. *kilimandscharica* to be sister to two *Macadamia* species, *Macadamia integrifolia* and *Macadamia ternifolia* with strong node support (Figure 1).

**Figure 1.**
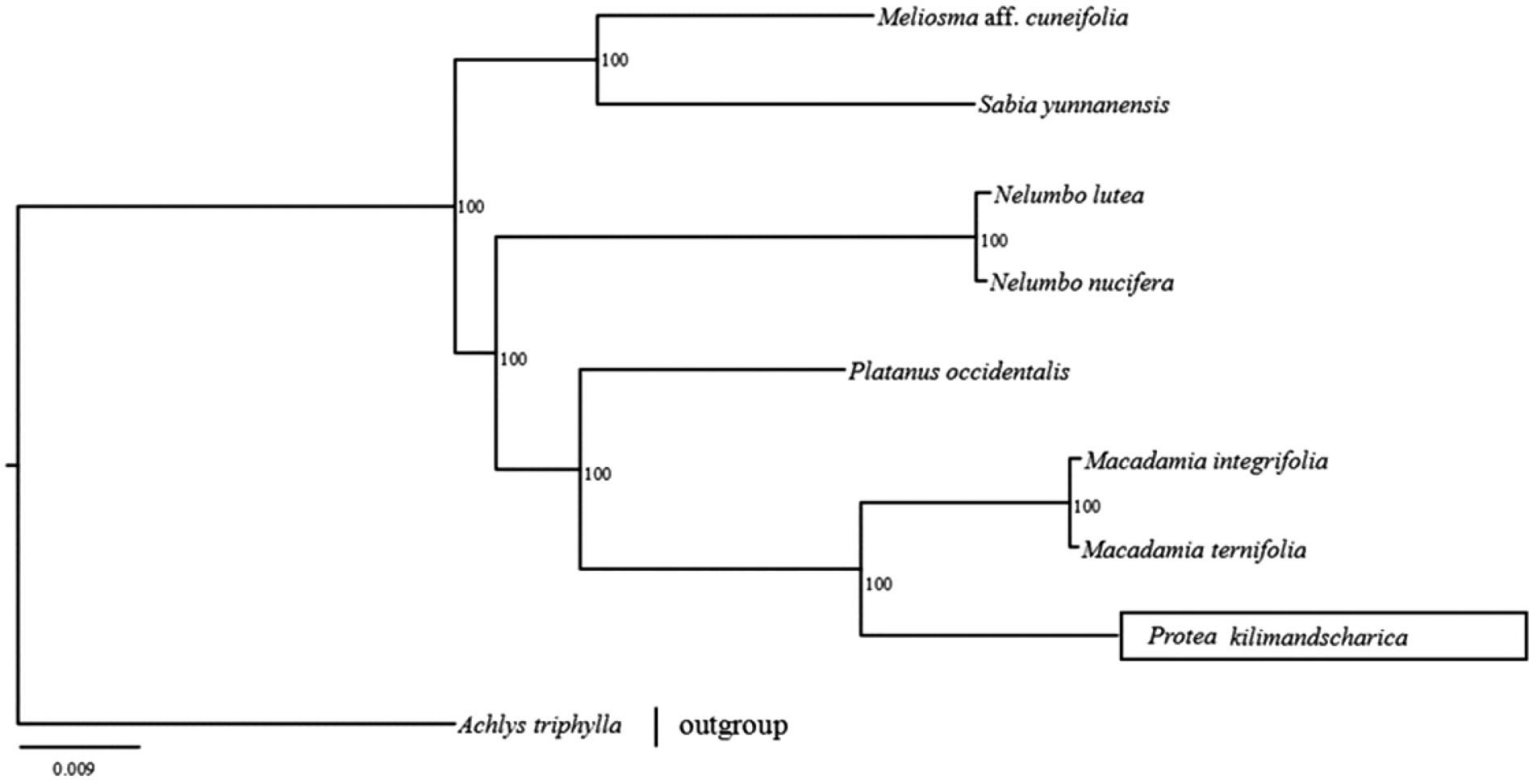
The maximum likelihood (ML) phylogenetic relationship of *Protea kilimandscharica* with related species based on 75 protein-coding genes common to the nine chloroplast genomes. The values at the nodes indicate the bootstrap support values. Accession numbers: *Protea kilimandscharica* MH362765*, Macadamia integrifolia* KF862711*, Macadamia ternifolia MF678834, Nelumbo nucifera* KF009944*, Nelumbo lutea* NC-015605*, Platanus occidentalis* NC_008335*, Sabia yunnanensis* NC_029431*, Meliosma cuneifolia* NC_029430*, Achyls triphylla* MG593050.
